# Quantitative Comparative Analysis of the Bio-Active and Toxic Constituents of Leaves and Spikes of *Schizonepeta tenuifolia* at Different Harvesting Times

**DOI:** 10.3390/ijms12106635

**Published:** 2011-10-10

**Authors:** Sheng Yu, Yiwen Chen, Li Zhang, Mingqiu Shan, Yuping Tang, Anwei Ding

**Affiliations:** Jiangsu Key Laboratory for High Technology Research of TCM Formulae, Nanjing University of Chinese Medicine, Nanjing 210046, China; E-Mails: yusheng1219@sina.com (S.Y.); owenchenlin@163.com (Y.C.); shanmingqiu@163.com (M.S.); yupingtang@njutcm.edu.cn (Y.T.)

**Keywords:** *Schizonepeta tenuifolia*, menthone, pulegone, limonene, menthofuran, harvesting time

## Abstract

A GC-MS-Selected Ion Monitoring (SIM) detection method was developed for simultaneous determination of four monoterpenes: (−)-menthone, (+)-pulegone, (−)-limonene and (+)-menthofuran as the main bio-active and toxic constituents, and four other main compounds in the volatile oils of *Schizonepeta tenuifolia* (ST) leaves and spikes at different harvesting times. The results showed that the method was simple, sensitive and reproducible, and that harvesting time was a possible key factor in influencing the quality of ST leaves, but not its spikes. The research might be helpful for determining the harvesting time of ST samples and establishing a validated method for the quality control of ST volatile oil and other relative products.

## 1. Introduction

The dried aerial part of *Schizonepeta tenuifolia* (Benth.) Briq. (ST, also called Jingjie in China) has long been popularly employed in mainland China, Hong Kong, Taiwan and Japan as a traditional medicinal herb for the treatment of colds, headaches, fevers, skin rashes (pruritus and rubella) and other disorders [1]. Much research regarding pharmacological actions *in vivo* and *in vitro* has shown its extracts (aqueous or methanolic) to have various biological and pharmaceutical properties, including antipruritic [2], hemostatic activity [3,4], and effects on the immune system [5,6]. The volatile components were recognized as the major constituents responsible for its biological effects. The volatile oil distilled from its aerial part and spike showed potent anti-inflammatory [7] and fumigant activity [8–10].

The volatile oils of ST contain many mono-terpenoids, including (−)-menthone, (+)-pulegone, (−)-limonene and (+)-menthofuran [11]. (−)-Menthone has been used as a fragrance in household products, components of artificial volatile oils, and tooth-brushing powder [12]. (−)-Limonene is one of the most common mono-terpenes in nature. It is a major constituent in several citrus oils (orange, lemon, mandarin, lime, and grapefruit). (−)-Limonene was listed in the Code of Federal Regulations of the USA as generally recognized as safe for a flavoring agent and can be found in common food items such as fruit juices, soft drinks, baked goods, ice cream, and pudding. (−)-Limonene was considered to have fairly low toxicity [13]. (+)-Pulegone is a natural mono-terpene obtained from the volatile oils of a variety of plants. It was used in flavoring agents, perfumery and aromatherapy. However, a recent study showed that it could cause severe hepatotoxicity, and its metabolism generated *p*-cresol; a glutathione depletory [14]. (+)-Menthofuran is a toxic furan terpenoid from various mint oils that was oxidized by mammalian cytochrome P450 to hepatotoxic metabolites. Evidence has been presented that *p*-cresol and other unusual oxidative products were metabolites of (+)-menthofuran in rats and that *p*-cresol might be responsible in part for the hepatotoxicity caused by (+)-menthofuran [15]. In addition, 1-octen-3-ol, 3-octanone, β-myrcene and β-caryophyllene were also main constituents of ST volatile oil, which had different biological activity. 1-Octen-3-ol and 3-octanone were both typical “moldy” odorants [16]; β-myrcene had a potent hepatoprotective action [17]; and β-caryophyllene was a functional cannabinoid receptor (type 2) agonist [18].

Many Chinese Patent Medicines were manufactured by using commercially available ST volatile oil or steam-distilled volatile oil from herb as a raw material. Their quality and content of bioactive component are influenced significantly by the climate, the cultivation conditions and even the time of harvesting and processing methods. In order to ensure stable quality of the crude herbs, many companies have adopted the good agricultural practice (GAP) for traditional Chinese medicine. In the process of establishing GAP of ST, the time of harvesting could not be determined because no relative data existed about the content variation trend of bioactive and toxic constituents in ST volatile oil over time.

So, the aim of this study was to comparatively evaluate the differences in the content of four monoterpenes including (−)-menthone, (+)-pulegone, (−)-limonene and (+)-menthofuran as the main bio-active and toxic constituents, and four other main compounds in the volatile oils of *S. tenuifolia* leaves and spikes at different harvesting times by using the gas chromatography-mass spectroscopy-selected ion monitoring detection method (GC–MS–SIM). Additionally, this research could be helpful to provide a validated method for the quality control of ST volatile oil.

## 2. Material and Methods

### 2.1. Plant Materials

The aerial parts of *Schizonepeta tenuifolia* were obtained at seven different harvesting times (listed in Table 1) from a Good Agriculture Practice (GAP)-based herbal garden which is located in Anguo, Hebei province. Herbs were then authenticated by Professor Qinan Wu, the botanist of the College of Pharmacy at Nanjing University of Chinese Medicine (Nanjing, China). Voucher specimens were also deposited at the herbarium at the above location. Plant materials were carefully cleaned and separated into two or three parts: leaf, stem and spike (if it existed), then a clevenger apparatus method was employed for the steam-distillation of volatile oils from separated leaf and spike parts. About 60 g of dried leaves or spikes were used for every sample.

### 2.2. Chemicals and Reagents

**Figure 1:**
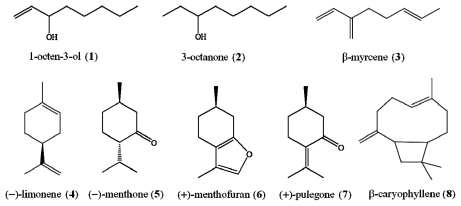
The chemical structures of eight compounds identified in ST volatile oil.

Ethyl acetate (gradient grade), *n*-decane (GC grade), naphthalene (GC grade) were purchased from Sinopharm Chemical Reagent (Shanghai, China). Chemical standards of 3-octanone, β-myrcene, (+)-menthofuran, and were purchased from Sigma-Aldrich (Vienna, Austria); (−)-menthone and (+)-pulegone were purchased from National Institutes for Food and Drug Control (Beijing, China); 3-octen-1-ol, (−)-limonene and β-caryophyllene were purchased from Tokyo Chemical Industural Co., Ltd (Tokyo, Japan). Their structures are presented in Figure 1.

### 2.3. Preparation of Sample and Standard Solutions

Volatile oil samples were extracted by water distillation for 4 hours from 60 g of ST leaf and spike, respectively, using a set of standard apparatus, according to the procedure described in the Pharmacopoeia of the Peoples’s Republic of China [19]. The volatile oils were isolated from water and dried over anhydrous sodium sulphate until the last traces of water were removed and then stored in the dark glass bottles at −20 °C. Fifty milligrams of the volatile oil sample were accurately weighed and transferred into a 10 mL volumetric flask, and 1 mL solutions of the two internal standards (containing 1.727 mg/mL of naphthalene and 0.252 mg/mL of *n*-decane in ethyl acetate) were transferred into the same flask, respectively, and made up to volume with ethyl acetate.

A mixed standard stock solution containing the eight reference compounds was prepared in ethyl acetate. The concentration of each compound in the stock solution was 284.64 μg/mL for 1-octen-3-ol (**1**), 53.24 μg/mL for 3-octanone (**2**), 100.12 μg/mL for β-myrcene (**3**), 802.80 μg/mL for (−)-limonene (**4**), 3.990 mg/mL for (−)-menthone (**5**), 24.42 μg/mL for (+)-menthofuran (**6**), 7.389 mg/mL for (+)-pulegone (**7**) and 157.28 μg/mL for β-caryophyllene (**8**), respectively. Working standard solutions were prepared by diluting the mixed standard stock solution with ethyl acetate to give six different concentrations within the ranges: (**1**), 5.7–227.7 μg/mL; (**2**), 1.1–42.6 μg/mL; (**3**), 2.0–80.1 μg/mL; (**4**), 16.1–642.2 μg/mL; (**5**), 79.8–3191.7 μg/mL; (**6**), 0.5–19.5 μg/mL; (**7**), 147.8–5911.0 μg/mL and (**8**), 3.1–125.8 μg/mL for calibration curves. 1.0 mL of double internal standards was also added into each solution of calibration.

All of the sample and standard solutions were filtered through a 0.45 μm membrane prior to injection. They were stored in a refrigerator at 4 °C before analysis.

### 2.4. GC-MS-SIM Analysis

**Figure 2:**
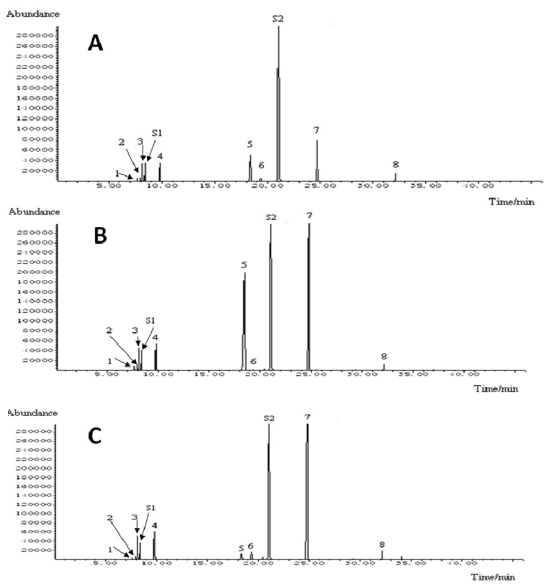
GC-MS-SIM Chromatogram of standard solution (**A**), volatile oils of ST leaves (**B**), volatile oils of ST spikes (**C**). Peak (**1**): 1-octen-3-ol; (**2**): 3-octanone; (**3**): β-myrcene; (**4**): (−)-limonene; (**5**): (−)-menthone; (**6**): (+)-menthofuran; (**7**): (+)-pulegone; (**8**): β-caryophyllene; IS1: *n*-decane; IS2: naphthalene.

1.0 μL of assayed solution was injected into Agilent 6890N (Agilent Technology, Austin, TX, USA) gas chromatograph (consisting of a 5975B mass selective detector and Agilent chemstation software package) equipped with an auto sampler (split ratio; 20:1). The analysis GC column was an HP-5MS (5% Phenyl Methyl Siloxane) capillary column (30 m × 0.25 mm ID, 0.25 μm film thickness). Helium (He) was the carrier gas at a flow rate of 1.0 mL/min. The GC temperature program was as follows: initial temperature was 50 °C, increased to 90 °C at a rate of 10 °C/min, at the temperature maintained for 5 min; then increased to 160 °C at a rate of 10 °C/min and held for 10 min. The injection temperature was 220 °C, transfer line temperature was 280 °C, and ion source temperature was 230 °C. The mass spectrometer was operated at 70 eV in the electron impact mode with selected ion monitoring (SIM) mode. The selected ion groups for the identification of eight compounds in SIM mode were listed in Table 2. The dwell time for each ion was set at 50 ms. *n*-decane and naphthalene were used as two internal standards (IS) for quantification. The GC-MS-SIM chromatograph of eight compounds was shown in Figure 2. The chemical identities of the resulting components were determined by matching their recorded mass spectra with the data bank mass spectra (NIST libraries) provided by the instrument software. The structures of the components were further confirmed by authentic standards analyzed under the conditions mentioned above.

### 2.5. Validation of the GC-MS-SIM Method

The dilute solution of the reference compounds was further diluted to a series of concentrations with ethyl acetate to assess the limits of detection (LOD) and quantification (LOQ). The LOD and LOQ were determined as signal-to-noise (S/N) ratios of 3 and 10, respectively. The intra- and inter-day precision was determined by analyzing calibration samples during a single day and on three consecutive days, respectively. To confirm the repeatability, five different working solutions were analyzed. The R.S.D. was taken in order to measure precision and reproducibility. The recovery test was used to evaluate the accuracy of this method. In the test, reference compounds were added to the L3 sample, and then analyzed as described above. The average recoveries were estimated by the formula: recovery (%) = (amount found − original amount)/amount added × 100%, and R.S.D. (%) = (S.D./mean) × 100%. For determination of the eight compounds, a calibration curve with double internal standards for each marker was constructed and tested for linearity. Their regression equations were calculated in the form of *Y* = A*X* + B, where *Y* and *X* (μg/mL) were peak area ratio of each component to the internal standard and compound concentration injected, respectively.

## 3. Results and Discussion

### 3.1. Identification of Chemical Components in the Volatile Oils

GC-MS-SIM Chromatogram of volatile oils from ST leaves and spikes, were shown in Figure 2. All the main components were separated completely in 35 min, and the eight compounds for quantification were identified on the basis of comparison of their mass spectra with NIST05 database through MSD ChemStation D.05.01, and with mass spectra of standard compounds, respectively. The results were listed in Table 2, and the structures of the identified compounds were shown in Figure 1.

### 3.2. GC-MS-SIM Method Validation

The proposed chromatographic method was validated to determine the linearity, LOD, LOQ, intraday and interday precisions, and accuracy. All calibration curves showed good linearity (*r*^2^ > 0.9990) within relatively wide concentration ranges, and the overall LODs and LOQs were less than 12.5 and 41.7 μg/mL, respectively (Table 3). The intra- and interday variations, repeatability, and stability RSD values of the eight compounds were all <2.37% and were shown in Table 4. The overall recoveries fell between 97.50 and 105.45% for the eight reference compounds, with RSDs of <2.76% (Table 5), which indicated that the established method was accurate enough for the determination of the eight compounds in the ST volatile oil samples.

### 3.3. Quantification of the Eight Compounds

The established GC-MS-SIM method was then subsequently applied to the simultaneous determination of the eight compounds in the volatile oil samples collected at different harvesting times. The assay results of eight compounds in volatile oils were summarized in Table 6. (+)-Pulegone and (−)-menthone, two *p*-menthane monoterpenes (also found in other aromatic plants in the Lamiaceae, probably due to the same biosynthetic pathway [20]) were determined as being the predominant constituents of the ST volatile oil, accounting for 85%~95% of total oils. Harvesting period (around flowering time) was a key stage for the biosynthesis of menthone in *S. tenuifolia*, in which pulegone was rapidly converted into menthone in leaves under regulation of (+)-pulegone reductase. Meanwhile, such bioconversion occurred much slower in spikes. (+)-pulegone was also found to be the single dominant constituent of spikes.

In Table 6, the content of (−)-menthone, an important bio-active constituent, increased from the growing stage (in late August) to the stage of fruitage (in early October) in ST leaves. However, it decreased from the spikes’ formation stage (in early September) to the stage of fruitage in ST spikes. The content of (−)-limonene, another bio-active constituent, did not vary much with harvesting time in ST leaves or spikes. The content of (+)-pulegone, a main toxic constituent, obviously decreased from the growing stage to the stage of fruitage in ST leaves, but it showed a slightly increasing trend from the spikes’ formation stage to the stage of fruitage in ST spikes. The content of (+)-menthofuran, another toxic compound, had virtually the same variation trends in ST leaves and spikes. The contents of other four compounds for quantification remained relative stable during the different harvesting times.

All of the results demonstrated that the optimum harvesting time of ST leaves was possibly in the stage of fruitage (in early October), and the optimum harvesting time of ST spikes was possibly in the spikes’ formation stage (in early September). However, the content of (+)-pulegone, only one dominant constituent of ST spikes, showed a very slightly increasing trend during the harvesting times. Therefore, harvesting time is possibly not a key factor for influencing the quality of ST spikes.

## 4. Conclusions

This is the first report about the simultaneous determination of eight main constituents in ST oils by using each authentic standard compound as a reference. The developed method was simple, sensitive and reproducible. Based on the analytical method, the quantitative comparative analysis was performed for the bio-active and toxic constituents of ST leaves and spikes at different harvesting times. The results showed that harvesting time was a key factor in influencing the quality of ST leaves, but not its spikes, which would be helpful for determining the harvesting time of ST samples and further establishing GAP of ST to ensure the quality of ST products.

## Figures and Tables

**Table 1:** Harvesting times of ST samples. L1~L7: *Schizonepeta tenuifolia* (Benth.) Briq. (ST) leaf samples; S3–S7: ST spike samples.

Samples No.	Harvesting time
L1	Growing stage (August 25, 2009)
L2	Formation of spikes (September 2, 2009)
L3, S3	Formation of spikes (September 9, 2009)
L4, S4	Full bloom (September 16, 2009)
L5, S5	Full bloom (September 23, 2009)
L6, S6	Stage of fruitage (September 30, 2009)
L7, S7	Stage of fruitage (October 6, 2009)

**Table 2:** The identification of eight compounds of ST volatile oils in GC-MS-Selected Ion Monitoring (SIM) mode.

No.	Compound	RT/min	MW	Formula	Selected ion group	Internal standard
1	1-octen-3-ol	7.66	128.21	C_8_H_16_O	43, 99, 128	*n*-decane
2	3-octanone	7.93	129.21	C_8_H_14_O	72, 99, 128,	*n*-decane
3	β-myrcene	8.12	136.23	C_10_H_16_	27, 41, 93, 136	*n*-decane
4	(−)-limonene	9.8	136.24	C_10_H_16_	69, 79, 136	*n*-decane
5	(−)-menthone	18.46	154.25	C_10_H_18_O	69, 112, 139, 154	naphthalene
6	(+)-menthofuran	19.35	150.22	C_10_H_14_O	79, 108, 150	naphthalene
7	(+)-pulegone	24.76	152.23	C_10_H_16_O	105, 112, 137, 152	naphthalene
8	β-caryophyllene	32.15	204.36	C_15_H_24_	105, 120, 133, 204	naphthalene

**Table 3:** Calibration curves and limits of detection (LOD) and limits of quantification (LOQ) data of the investigated compounds.

Analyte	Calibration curve	*r*^2^	Liner range (ng)	LOD (ng)	LOQ (ng)
1-octen-3-ol	*y* = 2.7312*x* − 7.3567	0.9998	5.7–227.7	1.0	2.8
3-octanone	*y* = 18.155*x* − 4.5699	0.9998	1.1–42.6	0.4	1.1
myrcene	*y* = 46.038*x* − 11.073	0.9998	2.0–80.1	0.8	2.0
(−)-limonene	*y* = 7.3369*x* + 51.41	0.9992	16.1–642.2	6.7	16.1
(−)-menthone	*y* = 0.3547*x* + 6.1814	0.9992	79.8–3191.7	2.5	8.3
(+)-menthofuran	*y* = 3.6063*x* − 0.4994	0.9996	0.5–19.5	0.2	0.5
(+)-pulegone	*y* = 0.1784*x* + 3.7122	0.9990	147.8–5911.0	12.5	41.7
β-caryophyllene	*y* = 0.5941*x* − 0.5272	0.9998	3.1–125.8	0.9	3.1

**Table 4:** Precision and repeatability stability of the eight analytes.

Analytes	Precision		Repeatability (RSD, %, *n* = 6)	Stability (RSD, %, *n* = 6)
	Intra-day (*n* = 6) RSD (%)	Inter-day (*n* = 3) RSD (%)		
1-octen-3-ol	0.46	0.32	1.51	0.65
3-octanone	0.09	1.21	2.68	0.70
myrcene	0.05	1.53	0.73	0.04
(−)-limonene	0.13	2.28	2.37	0.09
(−)-menthone	0.26	1.66	1.79	0.06
(+)-menthofuran	0.42	0.29	2.24	1.44
(+)-pulegone	0.16	1.87	1.92	0.40
β-caryophyllene	0.18	1.33	2.00	1.22

**Table 5:** Recovery of the eight analytes.

Analytes	Original (μg)	Added (μg)	Found (μg)	Recovery (%)	RSD (%, *n* = 6)
1-octen-3-ol	341.5	364.7	697.1	97.50	1.12
3-octanone	56.2	62.0	120.5	103.79	1.25
myrcene	110.4	117.4	231.4	103.11	1.73
(−)-limonene	947.2	885.9	1852.2	102.16	2.56
(−)-menthone	6026.4	5813.9	12157.4	105.45	1.50
(+)-menthofuran	26.4	49.1	76.8	102.75	2.76
(+)-pulegone	16862.6	16693.6	34031.6	102.85	2.47
β-caryophyllene	319.5	340.7	666.1	101.75	1.28

**Table 6:** Contents of eight investigated compounds in ST leaves and spikes (mg/g, *n* = 3).

Samples	Oil yield (g/g)	1	2	3	4	5	6	7	8
**L1**	0.042	0.39	0.08	0.27	2.11	7.04	0.05	30.55	0.44
**L2**	0.044	0.65	0.10	0.28	2.07	7.90	0.05	32.35	0.44
**L3**	0.044	0.60	0.10	0.19	1.76	10.61	0.05	29.68	0.56
**L4**	0.036	0.51	0.07	0.17	1.40	9.39	0.02	24.02	0.37
**L5**	0.033	0.49	0.08	0.17	1.41	9.33	0.01	20.89	0.35
**L6**	0.036	0.49	0.09	0.18	1.53	12.49	0.01	18.25	0.34
**L7**	0.037	0.52	0.10	0.19	1.57	16.74	0.01	14.66	0.37
**S3**	0.050	0.34	0.07	0.22	2.61	0.95	0.02	41.86	0.55
**S4**	0.048	0.31	0.06	0.23	2.27	0.82	0.03	41.49	0.50
**S5**	0.052	0.34	0.07	0.29	2.57	0.78	0.12	45.41	0.35
**S6**	0.048	0.31	0.06	0.29	2.41	0.66	0.14	42.51	0.36
**S7**	0.048	0.30	0.07	0.33	2.54	0.54	0.15	42.84	0.34
